# Fetal ciliopathies: a retrospective observational single-center study

**DOI:** 10.1007/s00404-021-06265-7

**Published:** 2021-10-01

**Authors:** Corinna Simonini, Anne Floeck, Brigitte Strizek, Andreas Mueller, Ulrich Gembruch, Annegret Geipel

**Affiliations:** 1 Department of Obstetrics and Prenatal Medicine, University Hospital Bonn, Venusberg-Campus 1, 53127 Bonn, Germany; 2 Department of Neonatology and Pediatric Intensive Care, University Hospital Bonn, Bonn, Germany

**Keywords:** Multisystem ciliopathy, Hyperechogenic kidneys, Meckel-Gruber syndrome, Jeune syndrome, Joubert syndrome, Bardet-Biedl syndrome

## Abstract

**Purpose:**

Report on the diagnosis of prenatally suspected multisystem ciliopathies in a single center between 2002 and 2020.

**Methods:**

Retrospective observational single-center study including pregnancies with prenatal ultrasound features of multisystem ciliopathies, such as hyperechogenic kidneys together with polydactyly and/or other skeletal and extraskeletal findings. Cases were compared according to their prenatal findings and outcomes.

**Results:**

36 cases of multisystem ciliopathies were diagnosed. Meckel-Gruber syndrome (MKS) was the most common ciliopathy (*n* = 19/36, 52.8%), followed by disorders that belong to the group of short-rib thoracic dysplasia (SRTD, *n* = 10/36, 27.8%) McKusick–Kaufmann syndrome (MKKS, *n* = 4/36, 11.1%), Bardet–Biedl syndrome (BBS, *n* = 2/36, 5.5%) and Joubert syndrome (*n* = 1/36, 2.8%). All cases showed abnormalities of the kidneys, most often hyperechogenic parenchyma (*n* = 26/36, 72.2%), cystic dysplasia (*n* = 24/36, 66.7%), and/or bilateral kidney enlargement (*n* = 22/36, 61.1%). Oligohydramnios was mainly present in fetuses with MKS. Polydactyly (*n* = 18/36), abnormalities of the CNS (*n* = 25/36), and heart defects (*n* = 10/36) were associated in 50%, 69.4%, and 27.8%, respectively.

**Conclusion:**

Prenatal detection of renal abnormalities associated with skeletal or brain abnormalities should raise the suspicion for multisystem ciliopathies. Prenatal ultrasound can help to differentiate between different diseases and pave the way for subsequent targeted genetic testing.

## Introduction

Ciliopathies form a broad spectrum of disorders with considerable phenotypic and genotypic overlap between different diseases and are caused by dysfunction of primary (or non-motile) cilia. Their estimated cumulative prevalence is 1 in every 2000 individuals [1]. Currently, we know of almost 190 causative genes and 35 established diseases, not least due to growing use of next-genome sequencing techniques [2]. Primary (or nonmotile) cilia are highly specialized cell organelles found on the surface of almost every mammalian cell type. There, as part of the so-called cilium-centrosome complex (CCC), they protrude from the cell’s surface and play a fundamental role in the detection and transduction of external signals to the interior of cells. Thus, dysfunction of primary cilia or the CCC can lead to complex diseases, often involving multiple organ systems [2, 3]. Autosomal dominant polycystic kidney disease (ADPKD), autosomal recessive polycystic kidney disease (ARPKD) and nephronophthisis (NPHP) were the first human single-gene ciliopathies described and are the leading representatives of inherited cystic kidney diseases. They share a predilection for the (hepato-)renal system whereas involvement of other organ systems is less frequent [4]. Many other ciliopathies, however, are characterized by the involvement of multiple organ systems as a result of the aforementioned ubiquitous presence of primary cilia. The spectrum of severity can range from mild with rather favorable prognosis (such as in Bardet–Biedl syndrome or McKusick–Kaufmann syndrome), to severe (such as in Joubert syndrome and related disorders), to fatal (such as in Meckel–Gruber syndrome or lethal skeletal ciliopathies) [5, 6].

Prenatal diagnosis of ciliopathies has become more frequent in the past 2 decades, however, still remains challenging because of their clinical and molecular heterogeneity as well as the considerable overlap of clinical features and causative genes [7]. The majority of publications covering the prenatal diagnosis of multisystem ciliopathies are case reports on individual diseases but, so far, there has not been published a comparison of different diseases based on the prenatal ultrasound findings.

The purpose of this study was to retrospectively analyze all cases of prenatally suspected and diagnosed multisystem ciliopathies other than ADPKD/ARPKD and NPHP in our center over the period of almost two decades. Special focus was put on individual prenatal ultrasound findings and outcome in order to assist in prenatal diagnosis and parent counselling in the future.

## Methods

This was a retrospective observational study including the data of more than 64,000 pregnancies booked at our tertiary level referral center of Obstetrics and Prenatal Medicine in Bonn, Germany, in the period between January 2002 and December 2020. Referrals to our center represent a mixed low- and high-risk population and are sent for targeted ultrasound examination or evaluation of suspected fetal anomalies. The majority of the cases presented here were sent for evaluation because of reduced amniotic fluid, urogenital abnormalities or both. All women received a detailed fetal anomaly scan including fetal echocardiography using high-resolution ultrasound equipment. We reviewed our prenatal ultrasound database for renal abnormalities from 2002 to 2020 (*n* = 2454), then filtered for cases of hyperechogenic and/or enlarged kidneys with or without cystic changes (*n* = 187). In this group, cases of underlying obstructive uropathy, unilateral kidney changes, or chromosomal abnormalities (*n* = 81) were excluded, which left us with a total number of 106 fetuses. Fetal multisystem ciliopathy was suspected based on the presence of abovementioned urogenital abnormalities (especially hyperechogenic kidneys) together with sonographic abnormalities of other organ systems (e.g., the central nervous system or the skeleton). Thus, cases of autosomal recessive or dominant polycystic kidney disease (ARPKD (*n* = 41), ADPKD (*n* = 6)) were also excluded. Some cases lacked image material or were lost to follow up and were therefor also excluded (*n* = 23). Extensive multidisciplinary counseling included information on further testing [i.e., genetic testing via chorionic villous sampling (CVS) or amniocentesis (AC)], pathophysiology, and course of the respective disease and the prognosis. Unfortunately, due to the length of the data acquisition and the prenatal methods used at that time, most cases were not evaluated according to the genetic standards used today.

We reviewed our data on the specific sonographic findings in different ciliopathies, genetic results as well as pregnancy outcome and long-term outcome of affected children, if available. For each case, outcomes were obtained from our perinatal database, neonatal records or autopsy findings. The research ethics board of Bonn University Hospital approved the study (ID 499/20).

## Results

Between January of 2002 and December of 2020, 36 fetuses were diagnosed with a disorder attributed to the ciliopathy spectrum, which gives an incidence of 6 in 10.000 pregnancies in our cohort. There was an equal female-to-male ratio (44.5% females, 47.2% males, sex unknown in 8.3%). Time of diagnosis is shown in Table 1.

**Table 1:** Characteristics of fetuses with prenatally diagnosed/presumed multisystem ciliopathies (*n* = 36)

Diagnosis	Mean GA at diagnosis (range)	Hyperechogenic kidneys	Other kidney abnormalities	Polydactyly	Abnormal CNS	(Other) sonographic findings	Amniotic fluid	Sex	Outcome
MKS (* n* = 19)	18 + 6 (11 + 0—30 + 5)	Yes (* n* = 18) No (* n* = 1)	Cystic kidney (* n* = 17, Fig. 1) Enlarged kidneys (* n* = 18, Fig. 1) Renal calcifications (* n* = 3) no CMD (* n* = 3)	Yes (* n* = 10) No (* n* = 9)	Yes (* n* = 18) No (* n* = 1)	Cardiac defects (* n* = 5) Others^●^ (* n* = 3)	Normal (* n* = 7)* Oligo-/Anhydramnios (* n* = 12)	Female (* n* = 4) Male (* n* = 12) Unknown (* n* = 3)	TOP (* n* = 18) Perinatal death (* n* = 1)
Jeune/SRTD (* n* = 10)	22 + 5 (15 + 4—30 + 3)	Yes (* n* = 5) No (* n* = 5)	Cystic kidney (* n* = 4) No CMD (* n* = 4) Enlarged kidneys (* n* = 3) Reverse CMD (* n* = 1) Hydronephrosis (* n* = 1)	Yes (* n* = 4) No (* n* = 6)	Yes (* n* = 4) No (* n* = 6)	Thoracic hypoplasia (* n* = 9) Micromelia (* n* = 9, Fig. 2) Curved tubular bones (* n* = 5, Fig. 2) Cardiac defects (* n* = 2) Others^●●^ (* n* = 3)	Normal (* n* = 6) Polyhydramnios (* n* = 3) Oligo-/Anhydramnios (* n* = 1)	Female (* n* = 6) Male (* n* = 4)	TOP (* n* = 8) Live-born (* n* = 2)
BBS / MKKS (* n* = 6)	30 + 1 (25 + 4—37 + 2)	Yes (* n* = 3) No (* n* = 3)	Hydronephrosis (* n* = 6, Fig. 3) Cystic kidney (* n* = 2, Fig. 3) Enlarged kidneys (* n* = 1)	Yes (* n* = 4) No (* n* = 2)	Yes (* n* = 2^●●●^) No (* n* = 1)	Hydrometrocolpos (* n* = 5, Fig. 3) LGA (* n* = 3) Anal atresia (* n* = 2) Cardiac defects (* n* = 3) Ascites (* n* = 1, Fig. 3) Others^●●●^ (* n* = 4, Fig. 3)	Normal (* n* = 4) Polyhydramnios (* n* = 1) Oligo-/Anhydramnios (* n* = 1)	Female (* n* = 6)	TOP (* n* = 1) NND (* n* = 1)** Live-born (* n* = 4)
Joubert (* n* = 1)	22 + 6	No (* n* = 1)	Solitary kidney cyst (* n* = 1, Fig. 4)	No (* n* = 1)	Yes (* n* = 1)	Molar tooth sign (Fig. 4), bilateral microophthalmia with ocular coloboma (Fig. 4)	Normal (* n* = 1)	Male (* n* = 1)	TOP (* n* = 1)

BBS = Bardet–Biedl syndrome; CMD = corticomedullary differentiation; CNS = central nervous system; GA = gestational age; LGA = large for gestational age; MKS = Meckel–Gruber syndrome, MKKS = McKusick–Kaufmann syndrome; NND = neonatal death; SRTD = short rib thoracic dysplasia (syndromes); TOP = termination of pregnancy;

^*^ Note: 5/7 cases of MKS with normal amount of amniotic fluid were diagnosed and terminated < 18 weeks of pregnancy;

^**^ cesarean section for severe preeclampsia at 29 + 6 weeks, neonatal death at day 1 due to severe prematurity, pulmonary hypoplasia and respiratory distress syndrome, postnatal examination confirmed vaginal and anal atresia

^●^Typical diagnostic triad (polycystic enlarged kidneys, occipital encephalocele and polydactyly) in 36.8% of all fetuses with MKS (*n* = 7/19); Other sonographic findings in fetuses with MKS: one anencephalus, one fetus with sacral-level spina bifida, one fetus with complete agenesis of the corpus callosum, asplenia, bifid uvula, arcuate uterus, hepatic fibrosis and pancreas divisum

^●●^ Other sonographic findings in fetuses with Jeune syndrome and related disorders: one fetus with thoracic/lumbar scoliosis, nuchal edema, hypertelorism and bilateral plexus cysts, one fetus with scoliosis and prefrontal edema, one fetus (with Mainzer–Saldino syndrome) with macrocephaly, hepatic cysts and fetal macrosomia

^●●●^Other sonographic findings in fetuses with BBS/MKKS: one fetus with double uterus and vagina (Fig. 4), one fetus with nuchal edema, clinodactyly of the fifth finger and hypertelorism, one fetus with bicornuate uterus; one fetus with clinically suspected BBS with heterotaxy syndrome (left atrial isomerism, situs ambiguus visceralis, interruption of the IVC with azygos continuation); CNS changes were observed only postnatally and were intracerebral calcifications in both cases

**Fig. 1:**
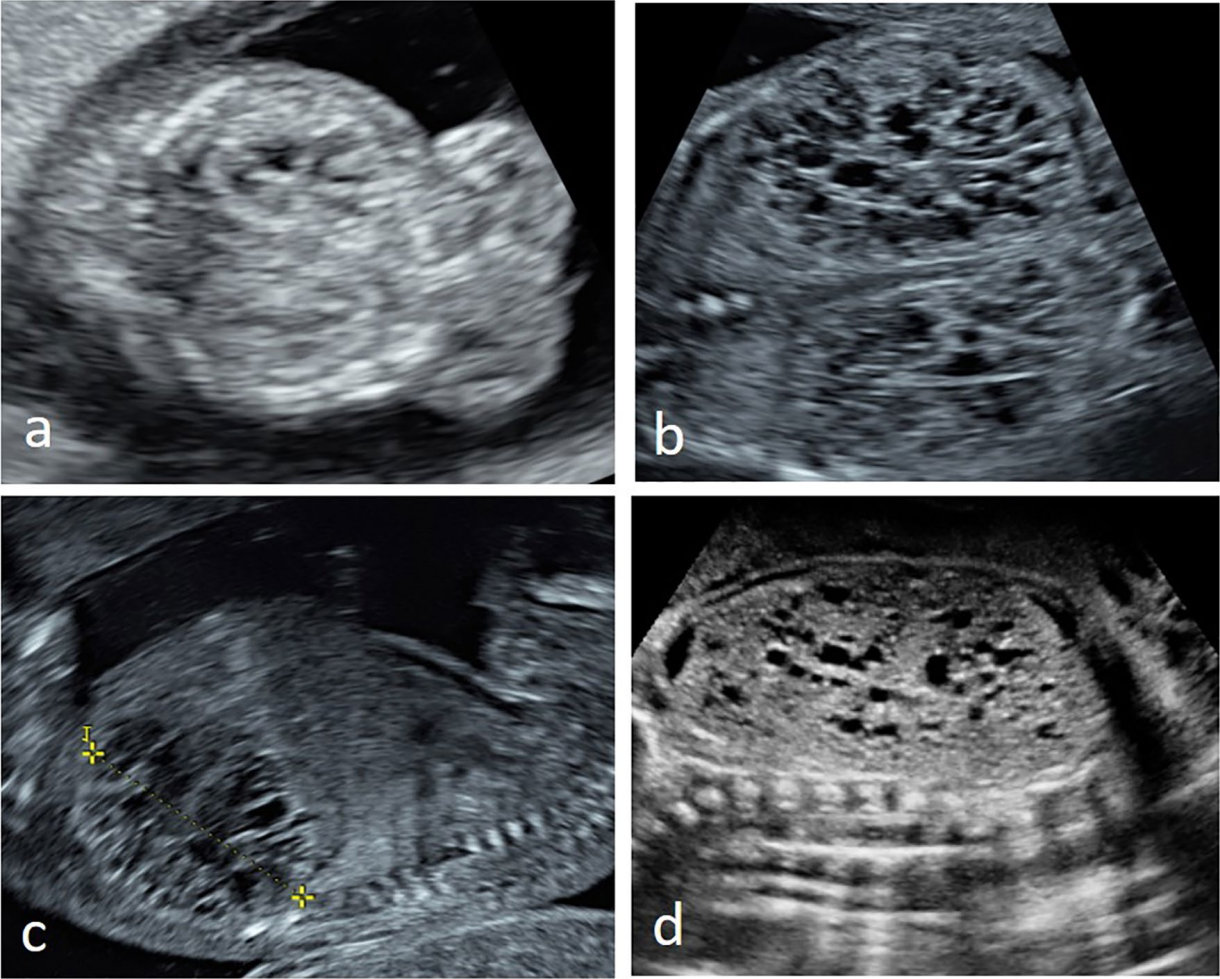
Prenatal ultrasound of typical polycystic and dysplastic kidneys in two fetuses with Meckel–Gruber syndrome at **a** 12 + 0 weeks and **b** + **c** at 14 + 5 weeks; note the premature (“too advanced”) CMD in first trimester and the missing CMD later in pregnancy; kidney cysts are small, numerous and intersperse in the entire renal parenchyma with pronounced occurrence in the medulla

**Fig. 2:**
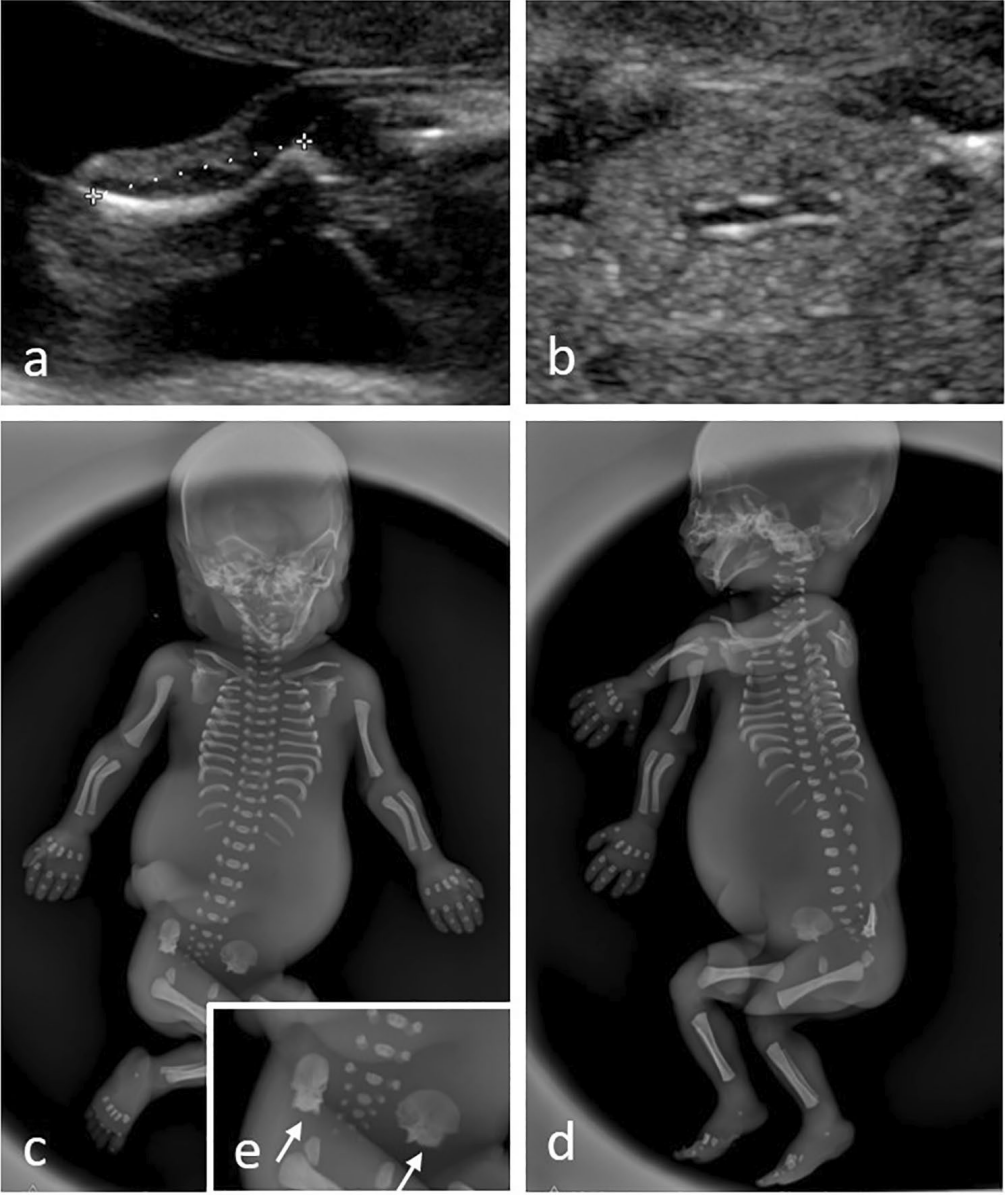
Pre- and postnatal (post-mortem) imaging in a fetus with Jeune syndrome: prenatal ultrasound showed **a** micromelia with a curved femur as well as **b** kidneys with missing corticomedullary differentiation at 21 + 3 weeks; **c**–**e **postnatal (post-mortem) radiography of the stillborn (TOP at 22 + 3 weeks): note the micromelia with the curved long bones as well as short ribs; **e** shows the typical “trident” appearance of the acetabular roof (white arrows)

**Fig. 3:**
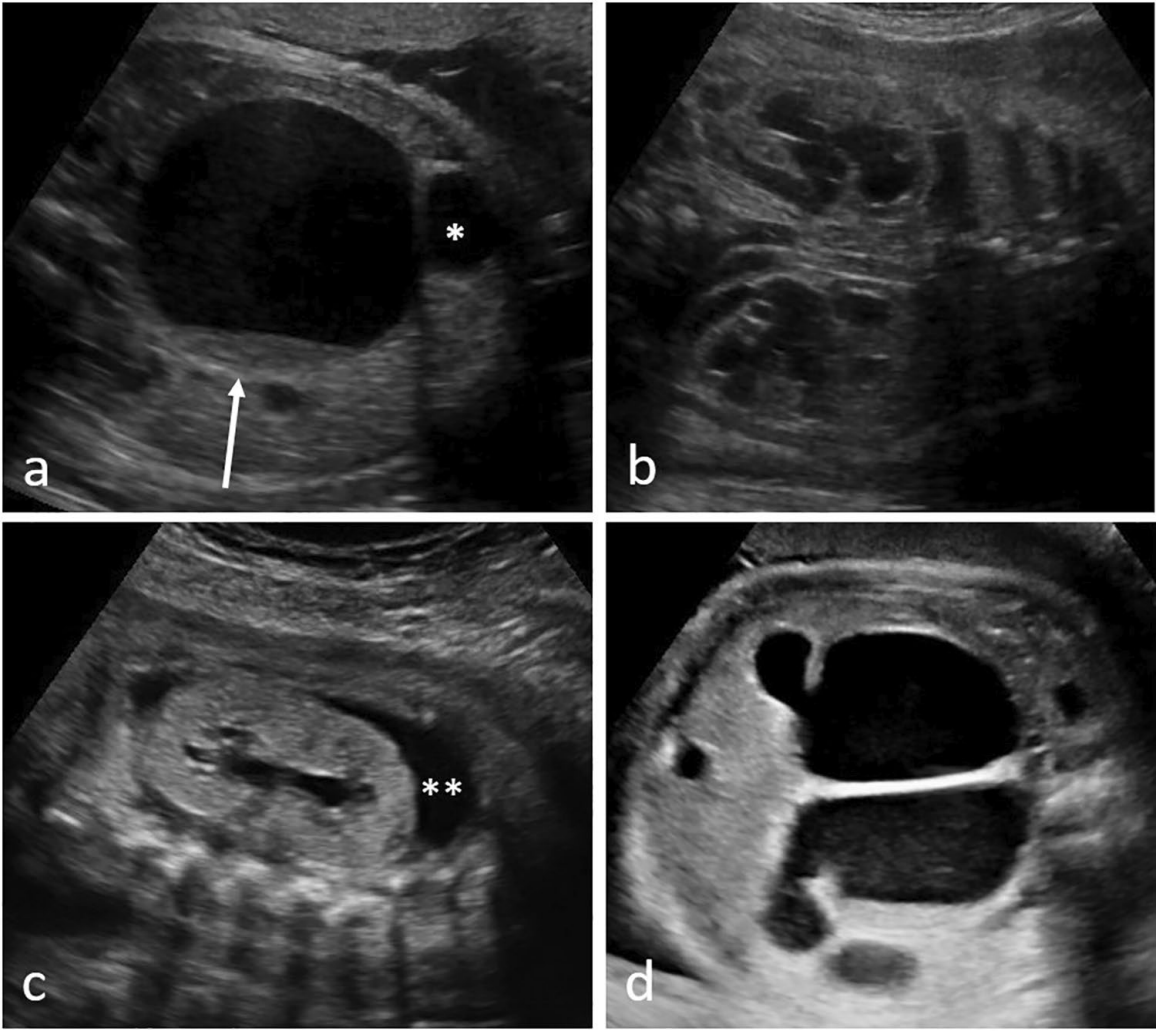
Prenatal ultrasound in three different fetuses with McKusick–Kaufmann syndrome: **a** fetus at 33 + 0 weeks showing hydrometrocolpos with sludge (arrow) as well as **b** bilateral hydronephrosis; *urinary bladder; **c** shows a different fetus at 26 + 0 weeks with hyperechogenic and dysplastic kidneys without corticomedullary differentiation and small cysts and **ascites; **d)** another fetus at 31 + 1 weeks showing hydrometrocolpos as well as double vagina and uterus

**Fig. 4:**
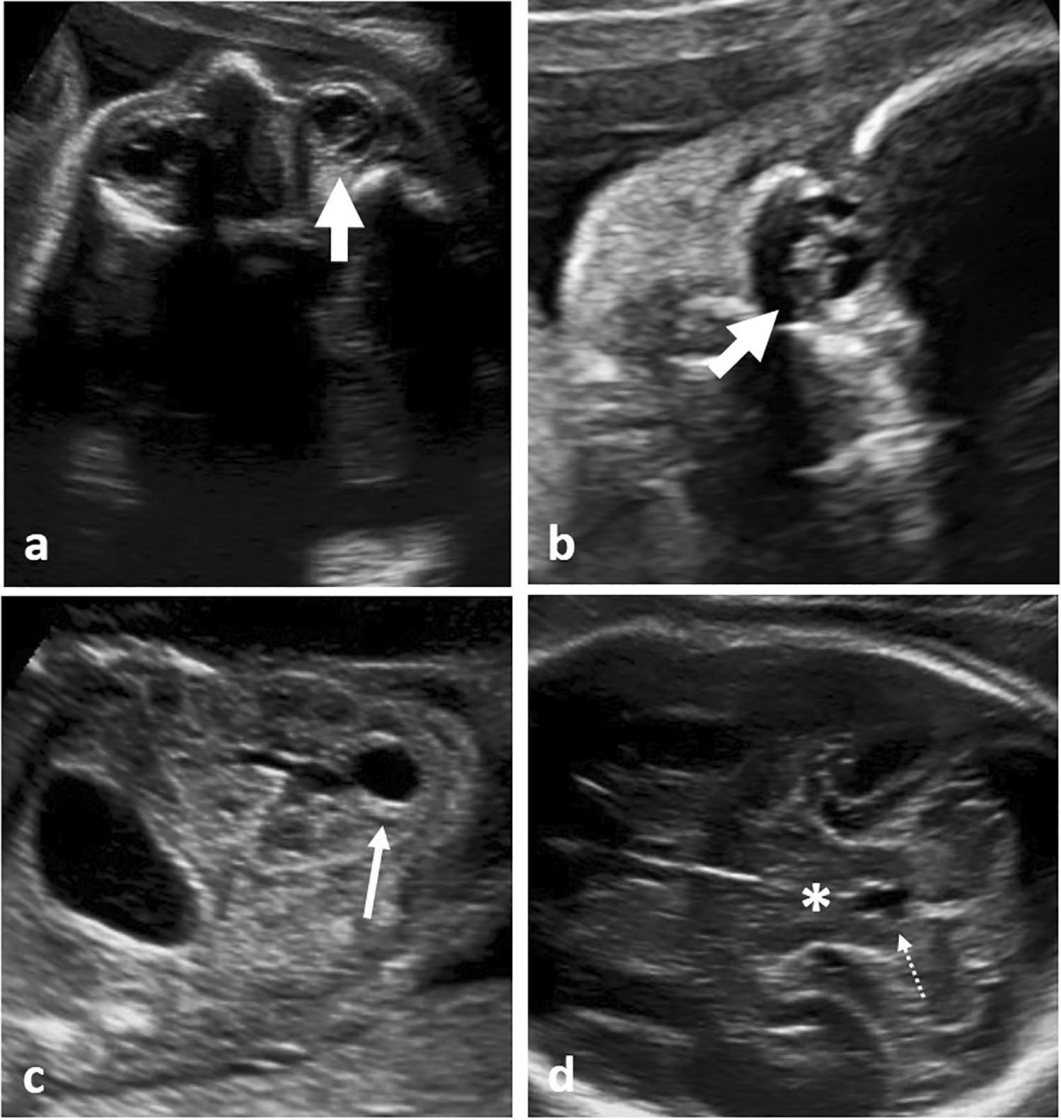
Prenatal ultrasound in a fetus with Joubert syndrome at 25 + 3 weeks; **a** bilateral microophthalmia with unilateral ocular coloboma (thick, short white arrow); **b** ocular coloboma (thick, short white arrow); **c** unilateral kidney cyst (thin white arrow), otherwise normal kidney structure; **d** molar tooth sign (*), note the elongated appearance of the fourth ventricle (thin, dotted white arrow)

Suspected diagnosis of a specific type of multisystem ciliopathy was made based on the presence of typical ultrasound findings, in some cases together with a positive medical history for a specific disease (*n* = 7/36; including 5 families with a history of Meckel–Gruber syndrome). The presumed diagnoses were confirmed either by genetic testing (*n* = 8), specific postpartum pediatric examination (*n* = 6), autopsy results (*n* = 8), or a combination of ultrasound findings, individual medical history and the clinical course of the pregnancy (*n* = 14).

Genetic testing was performed in 25 cases (69.4%, *n* = 23 prenatally, *n* = 2 postnatally, Table 2). Parental consanguinity was observed in 5 families (13.9%, Table 2). Successful genetic confirmation of the suspected disease was obtained in 8 cases (32%, Jeune syndrome and related disorders (*n* = 4), Meckel–Gruber syndrome (*n* = 2), and one case of Bardet–Biedl syndrome and Joubert syndrome, each).

**Table 2:** Genetic findings in fetuses / parents with prenatally diagnosed / presumed multisystem ciliopathies (*n* = 36)

Diagnosis	Consanguinity	Method of testing	Genetic results			Other
			**Fetus**	**Mother**	**Father**	
**Jeune/SRTD** **(*** n* **= 10)**	Unknown / no (* n* = 9) Yes (* n* = 1)	No genetic testing (* n* = 2) AC (* n* = 4) FBS (* n* = 2) CVS + AC (* n* = 1) Only parental testing (* n* = 1)	46, XX (* n* = 2) 46, XY (* n* = 2)			- One family with a history of 5 healthy children and 5 early miscarriages - One family with one prev. affected son (heterozygous mutation in *DYNC2H1 (c.10241C* > *A [p.Thr3414Asn]))*, one healthy daughter as well as another history of TOP for fetal thoracic hypoplasia and kidney abnormalities with normal karyotyping (46 XX, no further genetic testing) - One family with a history of 3 early miscarriages prior
			46 XX, compound heterozygous for 2 mutations in *DYNC2H1*; *c352C* > *T (p.Pro118Ser), c.10130delT (p.Leu3377CysfsX35)*	Heterozygous for mutations in *DYNC2H1*; *c352C* > *T (p.Pro118Ser)*	Heterozygous for mutations in *DYNC2H1*; *c.10130delT (p.Leu3377CysfsX35)*	
			No fetal testing	Heterozygous for pathogenic variants of *PKHD1* and *WDR60* Type 8	Heterozygous for pathogenic variants of *WDR60* and *DYNC2H1*	Consanguine couple
			46 XY; compound heterozygous for pathogenic mutations in *IFT140*; *c.454C* > *T p.(Leu152Phe)* + *c.1565G* > *A p.(Gly522Glu)*	Heterozygous for pathogenic mutation in *IFT140* *c.454C* > *T p.(Leu152Phe)*	Heterozygous for pathogenic mutation in *IFT140* *c1565G* > *Ap.(Gly522Glu)*	
			46 XY; compound heterozygous for pathogenic mutations in *IFT140*; *c.3109C* > T (pQ1037*)^●●●^ + *c.454C* > *T p.(Leu152Phe)*	-	-	
**Joubert** **(*** n* **= 1)**	Unknown (* n* = 1)	AC (* n* = 1)	46 XY, compound heterozygous for pathogenic mutations in *TMEM67*; *c.622A* > *T (p.Arg208Ter)* + *c.2498 T* > *C (p.lie833Thr)*	Heterozygous for pathogenic mutation in *TMEM67;* *c.622A* > *T*	Heterozygous for pathogenic mutation in *TMEM67;* *c.2498 T* > *C*	History of two healthy children (one son with VSD), 3 early miscarriages and one TOP for isolated encephalocele
**MKS** **(*** n* **= 19)**	Yes (* n* = 4) Unknown / no (* n* = 15)	No genetic testing (* n* = 8) AC (* n* = 5) CVS + AC (* n* = 1) CVS (* n* = 1) FBS (* n* = 3) Postnatal (* n* = 1)	46, XX (* n* = 2) 46, XY (* n* = 6)	-	-	- one family with a history of 2 healthy children and 1 early miscarriage (consanguine parents) - one family with a history of 9 healthy children, 1 early miscarriage (MKS), 3 TOPs for MKS (consanguine parents) - one family with a history of one TOP at 23 weeks for MKS as well as 2 healthy children (1 male, 1 female) - one family with a history of 3 healthy children and 2 early miscarriages with MKS - one family with a history of 1 prior son who had died of SCID/Omenn syndrome at the age of 3 months
			46, XY; homozygous deletion in *MKS3 (TMEM67)* *c.579.580del p.(Gly19IfsX13)*	-	-	1 healthy son prior
			46 XX; homozygous nonsense-mutation of CEP290 Exon 9 p.Arg205Ter (c.613C > T)	-	-	History of 1 early miscarriage prior
			46, XX; incidental finding of pericentric inversion of chromosome 9^●●^	-	-	History of 1 prior son who died of leucaemia at the age of 9 months
**BBS/MKKS** **(*** n* **= 6)**	Unknown (* n* = 5) No (* n* = 1)	No genetic testing (n = 1) AC (* n* = 2) FBS (* n* = 2) postnatal (* n* = 1)	46 XX (* n* = 4)			
			46, XX, compound heterozygous for one pathogenic frameshift mutation in *BBS7* and another presumably pathogenic frameshift mutation in *BBS7;* c.712_715del, p.Arg238Glufs*59; c.2023.2029del, p.Thr675Phefs*3	-	-	Two healthy children prior

Abbreviations (in alphabetical order): AC = amniocentesis; BBS = Bardet–Biedl syndrome, CVS = chorionic villous sampling; FBS = fetal blood sampling; MKKS = McKusick–Kaufmann syndrome; MKS = Meckel–Gruber syndrome; SCID = severe combined immunodeficiency; SRTD = short rib thoracic dysplasia (syndromes); TOP = termination of pregnancy; VSD = ventricular septal defect; ^●●^considered to be a variant of normal karyotype; ^●●●^ mutation has not been reported previously

Meckel–Gruber syndrome (MKS) was the most common ciliopathy (*n* = 19/36, 52.8%) in our cohort, followed by Jeune asphyxiating thoracic dystrophy (JATD) and related disorders that belong to the group of short-rib thoracic dysplasia (SRTD, *n* = 10/36, 27.8%) and 4 fetuses with McKusick–Kaufmann syndrome (MKKS, *n* = 4/36, 11.1%). There were two cases of Bardet–Biedl syndrome (BBS) and one case of Joubert syndrome.

## Ultrasound findings in fetuses with different multisystem ciliopathies

Ultrasound findings are shown in Table 1. Mean GA at diagnosis was lowest in fetuses with MKS (18 + 6 week’s, range 11 + 0 to 30 + 5) and highest in fetuses with MKKS/BBS (30 + 1 week’s, range 25 + 4 to 37 + 2). First trimester ultrasound was abnormal in 6 cases (16.7%, 1 case of Jeune syndrome, 5 cases of MKS). Comparison of multisystem ciliopathies with focus on prenatal ultrasound findings showed different key features.

### Kidney changes and amniotic fluid

Hyperechogenic kidneys were present in 94.7% (*n* = 18/19) of fetuses with MKS as well as in 50% of Jeune/SRTD (*n* = 5/10) and cases with BBS/MKKS (*n* = 3/6). Almost all fetuses with MKS additionally showed massive bilateral kidney enlargement (94.7%, *n* = 18/19) as well as significant degree of cystic kidney dysplasia (89.5%, *n* = 17/19, Fig. 1). On prenatal ultrasound, the impression of a premature or, given the gestational age, “too advanced” corticomedullary differentiation (CMD) arose. The cortex appeared hyperechogenic, the medulla hypoechogenic. In 2nd and 3rd trimester, however, fetuses with MKS showed no recognizable CMD. Here, both cortex and medulla presented hyperechogenic. Kidney cysts in fetuses with MKS were usually small, numerous, and interspersed the entire renal parenchyma with a pronounced occurrence in the medulla (Fig. 1). Fetuses with other multisystem ciliopathies tended to show only mild kidney enlargement, with either solitary or few cortical cysts. Changes of corticomedullary differentiation (absent or reversed) were more common in fetuses with Jeune/SRTD (50%, *n* = 5/10, Fig. 2) compared to all other groups. Combined abnormalities of the genitourinary system were the leading sign of fetuses with BBS/MKKS and involved hydrometrocolpos in 83% (*n* = 5/6, Fig. 3) as well as hydronephrosis in all cases (*n* = 6/6). The fetus with Joubert showed a solitary cortical kidney cyst with, otherwise, normal renal structure (Fig. 4).

Oligohydramnios on second trimester ultrasound occurred most frequent in fetuses with MKS (83.3%, *n* = 10/12 after 18 + 0 weeks’). Fetuses affected by other ciliopathies presented with either normal or increased amount of amniotic fluid.

### Abnormalities of the central nervous system (CNS)

Fetuses with MKS most commonly presented with occipital encephalocele (79%, *n* = 15/19), but also Dandy–Walker malformation in 31.6% (*n* = 6/19). Less common findings included spina bifida, agenesis of the corpus callosum, or anencephalus. The fetus with Joubert syndrome presented with the typical and pathognomonic “molar tooth sign” on prenatal ultrasound (Fig. 4), together with Dandy–Walker malformation and an arachnoid cyst. In addition to that, the fetus presented with bilateral microophthalmia and unilateral ocular coloboma (Fig. 4). Other multisystem ciliopathies were not associated with CNS anomalies.

### Skeletal findings

Fetuses with Jeune/SRTD presented with thoracic hypoplasia in 90% (*n* = 9/10) as well as severe micromelia in 90% (*n* = 9/10). Additional curving of tubular bones was present in 50% (*n* = 5/10) of all Jeune/SRTD cases (Fig. 2). Polydactyly was a frequent sign in all groups, and was observed in 67% of fetuses with BBS/MKKS (*n* = 4/6), 52.6% (*n* = 10/19) of MKS and 40% (*n* = 4/10) of fetuses with Jeune/SRTD.

### Other ultrasound findings

In addition to CNS abnormalities and skeletal malformations, cardiac defects were seen in almost 28% (*n* = 10/26) of all multisystem ciliopathies: in 50% of fetuses with BBS/MKKS (*n* = 3/6), 26.3% (*n* = 5/19) of fetuses with MKS and 20% of cases with Jeune/SRTD (*n* = 2/10). Abnormal growth pattern (large for gestational age) occurred in 3 out of 6 fetuses with verified BBS/MKKS.

Outcome data are given in Tables 1 and 3. There was termination of pregnancy (TOP) in 77.8% (*n* = 28/36), perinatal death in one case (MKS) and 7 infants were born alive (19.4%, *n* = 7/3). Survival beyond the neonatal period was 85.7% (*n* = 6/7; range of follow-up: 1 month to 16 years).

**Table 3:** Long-term outcome of survivors with prenatal diagnosis of multisystem ciliopathy (*n* = 6)

Diagnosis	GA at birth (weeks + days)	Sex	Birth weight (g) (percentiles)	Follow-up time	Long-term outcome
Jeune	30 + 2	Male	1435 (42.)	18 months	Home ventilation for pulmonary hypoplasia; mild nephrocalcinosis, death at the age of 18 months
Mainzer–Saldino syndrome	39 + 0	Male	4400 (> 97.)	6 months	Chronic renal insufficiency due to polycystic renal dysplasia, continuous peritoneal dialysis; continuous ventilation for pulmonary hypoplasia, hepatomegaly, liver cysts; pancreas cyst; progressive cystic remodeling of kidneys, liver and pancreas together with multiple abdominal cysts and ascites; death at the age of 6 months due to pulmonary hypertension
BBS	37 + 3	Female	3920 (> 97.)	9,5 years	Postnatal surgery for anal and vaginal atresia, correctional surgery for AV canal at the age of 10 months; current status: obesity, mental retardation (patient attends special-needs-school), chronic renal insufficiency stage III, recurrent urinary tract infections, mild urinary and fecal incontinence, brain: thalamostrial calcifications;
BBS*	40 + 1	Female	3400 (42.)	16 years	Postnatal surgery for polydactyly as well as malposition of the common bile duct; current status: vision impairment, obesity, mild mental retardation (attends special needs school), genetic testing was declined by the parents
MKKS/BBS	36 + 5	Female	3990 (> 97.)	1 month	Postnatal ultrasound confirmed vaginal atresia and bilateral hydronephrosis due to compression of both ureteres
MKKS	37 + 0	Female	4160 (> 97.)	8 years	Postnatal surgery for anal and vaginal atresia; current status: recurrent urinary tract infections, mild urinary and fecal incontinence, otherwise normal development

Abbreviations (in alphabetical order): AV canal = atrioventricular canal; BBS = Bardet–Biedl syndrome; CS = cesarean section; g = grams, GA = gestational age, MKKS = McKusick–Kaufmann syndrome; NICU = neonatal intensive care unit; VB = vaginal birth; * diagnosis “BBS” based on clinical findings

## Discussion

This study presents 36 cases of prenatally suspected and diagnosed multisystem ciliopathies with special focus on individual prenatal ultrasound findings and outcome in different diseases. So far, this is the largest single-center study focusing on the prenatal diagnosis of multisystem ciliopathies other than ARPKD/ADPKD and NPHP.

With an incidence of 2.6 per 100.000 reported in the literature [8], Meckel–Gruber syndrome (MKS, OMIM #249000) is one of the most frequent multisystem ciliopathies. The clinical diagnosis is usually made based on the typical triad of enlarged dysplastic and polycystic kidneys, occipital encephalocele, and postaxial hexadactyly, which, in our cohort, were given in 94.7%, 79%, and 52.6% of all MKS cases, respectively. This is consistent with current literature [8, 9]. However, the phenotypic appearance can vary [10, 11] even from pregnancy to pregnancy as some of our cases of families with repeated MKS showed. In our cohort, only 36.8% of fetuses showed all three classical features of the triad, which is comparable to other study findings [9]. Compared to other ciliopathies, MKS was significantly more associated with oligo- or anhydramnios in second and third trimester. Up to this point, more than 21 different causative genes have been identified in MKS [12], however, genetic testing often remains inconclusive. Given its unfavorable prognosis, correct diagnosis is important for optimal counselling of affected parents. As the typical sonographic abnormalities can be found from 10 weeks’ onwards, women with a history of MKS should receive a detailed first trimester scan.

Jeune syndrome (or “Jeune asphyxiating thoracic dystrophy”, OMIM #208500), and related disorders, all belong to the group of short-rib thoracic dysplasia (SRTD) with or without polydactyly. This broad group of skeletal ciliopathies (or “ciliary chondrodysplasias”) shares features like a constricted thoracic cage, short ribs, shortened tubular bones, brachydactyly and a “trident” appearance of the acetabular roof. An incidence of 1 in 100,000 to 130,000 live births has been reported [13]. Whereas Jeune syndrome is sometimes referred to as the “prototype” of skeletal ciliopathies, other common skeletal ciliopathies include Mainzer–Saldino syndrome (or “conorenal syndrome”, OMIM #266920), Ellis-van Creveld syndrome (or “chondroectodermal dysplasia”, OMIM #225500), Sensenbrenner syndrome (or “cranioectodermal dysplasia”, OMIM #218330), orofaciodigital syndrome 4 (OMIM #258860) and short-rib polydactyly syndromes (SRPSs) types 1–4 (OMIM #611263, #613091, #263520, #269860, #614091) [13].

Key ultrasound features of SRTD involve the skeletal system (thoracic hypoplasia, short tubular bones) with phenotype severity significantly varying between the different conditions, but also between patients with the same disease. Usually, the most severe thoracic constriction is seen in SRP type I–IV and “true” Jeune syndrome. Other skeletal ciliopathies, such as Mainzer–Saldino syndrome, might display a much milder phenotype with regards to thoracic hypoplasia, however, frequently suffer from other, extraskeletal, symptoms such childhood onset end-stage renal disease, liver disease and retinal degeneration [14, 15]. Degree and severity of renal changes in SRTD vary significantly and are usually much more subtle compared to MKS. In our cohort, these changes included hyperechogenic kidneys with absent or reversed CMD in 50% of all cases, followed by kidney cysts and mild kidney enlargement. Amount of amniotic fluid was normal or even increased in 90%. All these findings are consistent with current literature [16, 17]. Interestingly, our patient with Mainzer–Saldino syndrome, that died at the age of 6 months due to pulmonary hypertension, showed progressive cystic remodeling of the kidneys, liver and pancreas as well as spontaneously emerging intraabdominal cysts and ascites with continuously aggravating findings in the months after birth. In fetuses with SRTD, different parameters have been proposed in order to assess severity of pulmonary hypoplasia on prenatal ultrasound, such as the thoracic circumference/abdominal circumference (TC/AC) ratio, in order to counsel expecting parents about the individual prognosis [18]. The complexity of phenotypical overlapping of different skeletal ciliopathies on prenatal ultrasound further highlights the need for targeted genetic testing in order to define the individual genes affected and, in this way, predict the individual prognosis and counsel parents adequately [15].

McKusick–Kaufman syndrome (MKKS, OMIM #236700) and Bardet–Biedl syndrome (BBS, OMIM #209900) are two syndromes with significant clinical overlap and often these two conditions cannot be told apart prenatally or even in early infancy. MKKS is usually diagnosed on the basis of the typical diagnostic triad of hydrometrocolpos, postaxial polydactyly and congenital cardiac disease [19–21], which, in our cohort, were present in 83%, 67%, and 50%, respectively. Hydronephrosis was the leading renal abnormality and present in all our MKKS/BBS cases with either normal or enhanced amniotic fluid in 83% of all cases. Compared to other multisystem ciliopathies, average GA at diagnosis in fetuses with MKKS/BBS was significantly higher in our study cohort, however, detection of ultrasound abnormalities in fetuses with BBS as early as at 15 weeks has been described [19]. All our MKKS/BBS fetuses were female, but MKKS/BBS in males is possible and can be accompanied by genital malformations or hypogonadism. In addition to the genitourinary abnormalities, anal atresia was present in 33% (*n* = 2/6) of fetuses with MKKS/BBS in our cohort, which is also consistent with current literature [21]. MKKS is caused by mutations in the MKKS gene mapped onto chromosome 20p12, however, mutations in the same gene also cause Bardet–Biedl-6 syndrome (BBS-6, OMIM #209900), which, later in childhood, presents with additional retinitis pigmentosa, obesity, mental retardation and renal failure [22, 23]. Since MKKS patients usually do not develop retinopathy, learning disabilities or obesity later in life, optimal and targeted prenatal genetic testing is crucial for adequate counseling of future parents with regards to prognosis and optimal pediatric management [21]. Interestingly, 50% of our fetuses with BBS/MKKS presented as large for gestational age during pregnancy. The exact mechanisms underlying central obesity commonly found in patients with BBS are not fully clear yet (amongst others, impaired leptin receptor trafficking and signaling as well as impaired insulin signaling as a result of ciliary dysfunction have been proposed, [24]), but this finding might serve as a possible link from pre- to postnatal diagnosis. Studies in mouse models have raised suggestions that a complete absence of the MKKS gene leads to BBS (phenotypes) while the MKKS phenotype is likely to be due to specific mutations [25]. Other studies showed, that the domain deleted in the protein encoded by the Cep290^rd16^ allele (which, as we know, plays a role in Joubert Syndrome, Meckel–Gruber syndrome, Leber congenital amaurosis and BBS-14) directly interacts with the MKKS protein [26]. Again, this highlights the high frequency of genetic overlapping found in multisystem ciliopathies. Especially when prenatal genetic testing (even if targeted) is unrewarding, children with prenatally diagnosed MKKS should be reevaluated on a regular basis for signs of Bardet–Biedl syndrome during childhood and a final diagnosis should not be made before the age of 5 years [21, 23, 27].

Joubert syndrome (JS) and related disorders (JSRD) share an incidence of 1:80,000–100,000 live births. Pathognomonic for this specific ciliopathy is the so-called “molar tooth sign” on axial views of the brain (Fig. 4), which is caused by thickened superior cerebellar peduncles, cerebellar vermis hypoplasia, and a deepened interpeduncular fossa. Molar tooth sign, however, is not specific for Joubert syndrome and can be found in other (partially Joubert-related) ciliopathies as well [28–30]. According to the organ system mainly affected, subdivision of JS into six phenotypic subgroups has been proposed [31]: pure JS, JS with ocular defect, JS with renal defect, JS with oculorenal defects, JS with hepatic defect and JS with orofaciodigital defects. Other authors suggest classification into only two groups, JS with and without retinal dystrophy. Reason for this classification was the fact that renal cysts (usually multiple, small and cortical), liver cysts but also interstitial kidney fibrosis and damage without cysts could only be found in patients that showed retinal dystrophy. Our fetus also showed a solitary cortical cyst as well as ocular coloboma and an arachnoid cyst, making a predilection for cystic dysplastic changes of various organ tissues probable. Clinically, patients with JS show hypotonia as well as developmental delay, together with abnormal breathing and abnormal eye movements in a high number of cases. Prenatal diagnosis of JS/JSRD is rare as ultrasound signs can be subtle (see Fig. 4). Especially in fetuses lacking prenatal abnormalities other than of the cerebellum, differential diagnosis from Dandy–Walker malformation can be difficult. Saraiva and Baraitser [32] suggested that in JS different degrees of vermian dysplasia can be found and are usually most pronounced posteriorly and inferiorly. Additionally, hypoplasia of the brain stem can be present and its involvement could explain some of the symptoms presented by patients with JS (such as abnormal breathing pattern and eye movements). The research work by Bachmann-Gagescu et al. [33] as well as the review by Parisi et al. [34] further highlight the extreme genetic heterogeneity found in JS/JSRD.

We acknowledge that, aside from the general limitations of retrospective studies, the specific limitations of our study include small numbers in certain disease groups as well as the restrictions associated with a tertiary referral center which tend to see more severe cases and may therefore have higher rates of TOP as well as lower overall survival. In addition, data acquisition over a period of time of almost two decades with significant change of methods and availability of genetic testing are part of the reason for a fairly low rate of effective and successful genetic testing (32% in our study group) and most cases were not evaluated according to the genetic standards used today.

Concluding, multisystem ciliopathies represent a continuum of disorders rather than distinct and strictly separable diseases, and certain prenatal ultrasound findings, particularly the combination of renal abnormalities together with either skeletal and/or brain abnormalities, should raise the suspicion of ciliary dysfunction, especially when common aneuploidies such as trisomy 13 are excluded.

Based on the specific ultrasound findings and the parents’ medical history, targeted genetic testing (“precision medicine”) should always be offered in order to counsel affected parents adequately—not least to detect and sometimes prevent future medical problems of affected children [35]. Up to this day, clinicians and affected families are challenged by the considerable and often striking overlaps and heterogeneity found in multisystem ciliopathies and diagnostic rates of 62% using targeted gene panel sequencing and 44% using whole exome sequencing further emphasize that “giving the disease a name” is still not possible in a high number of cases [36]. Future studies with special focus on genotype–phenotype correlation in different diseases are needed in order to gain more knowledge on this complex disease spectrum.

## Data Availability

Not applicable.
